# Detrimental effects of rat mesenchymal stromal cell pre-treatment in a model of acute kidney rejection

**DOI:** 10.3389/fimmu.2012.00202

**Published:** 2012-07-17

**Authors:** Martina Seifert, Meaghan Stolk, Dietrich Polenz, Hans-Dieter Volk

**Affiliations:** ^1^Berlin-Brandenburg Center for Regenerative Therapies, Charité Universitätsmedizin Berlin, Berlin, Germany; ^2^Institute of Medical Immunology, Charité Universitätsmedizin Berlin, Berlin, Germany

**Keywords:** mesenchymal stromal cell, inflammation, kidney transplantation, acute rejection, humoral response

## Abstract

Mesenchymal stromal cells (MSC) have shown immunomodulatory and tissue repair potential including partial tolerance induction by pre-treatment of donor-specific cells in a rat heart transplantation model. Very recently, we could show that autologous MSC attenuated ischemia reperfusion injury in a highly mismatched donor–recipient rat kidney transplant model. Therefore, we investigated donor-specific MSC pre-treatment in this rat kidney transplantation model to study whether graft function could be improved, or if tolerance could be induced. Donor- and recipient-type MSC or phosphate buffered saline (PBS) as a control was injected i.v. 4 days before kidney transplantation. Mycophenolate mofetil immunosuppression (20mg/kg body weight) was applied for 7 days. Kidney grafts and spleens were harvested between days 8 and 10 and analyzed by quantitative RT-PCR and immunohistology. In addition, creatinine levels in the blood were measured and serum was screened for the presence of donor-specific antibodies. Surprisingly, application of both donor- and recipient-specific MSC resulted in enhanced humoral immune responses verified by intragraft B cell infiltration and complement factor C4d deposits. Moreover, signs of inflammation and rejection were generally enhanced in both MSC-treated groups relative to PBS control group. Additionally, pre-treatment with donor-specific MSC significantly enhanced the level of donor-specific antibody formation when compared with PBS- or recipient MSC-treated groups. Pre-treatment with both MSC types resulted in a higher degree of kidney cortex tissue damage and elevated creatinine levels at the time point of rejection. Thus, MSC pre-sensitization in this model impairs the allograft outcome. Our data from this pre-clinical kidney transplantation model indicate that pre-operative MSC administration may not be optimal in kidney transplantation and caution must be exerted before moving forward with clinical studies in order to avoid adverse effects.

## INTRODUCTION

Kidney transplantation outcomes have been greatly improved over the last few years by better immunosuppression regimens and post-operative care. However, due to organ shortages, often the donor kidneys available are sub-optimal or so-called “marginal organs,” which has been shown to lead to greater problems with immunogenicity and worse long-term function (Audard et al., 2008; Stallone et al., 2010). Several attempts have been developed to help reduce damage to the graft that may occur before the transplant (van der Woude et al., 2004; Kotsch et al., 2007; Caumartin et al., 2011), however, many treatment regimes are not suited for use with human patients. More recent strategies have focused on using cell therapies from different sources to help stimulate the regeneration of cells inside the transplanted organ (Bussolati and Camussi, 2006; Morigi et al., 2006; Choi et al., 2010; Harari-Steinberg et al., 2011; Little, 2011; Bussolati et al., 2012). In particular, the reduction of ischemia reperfusion injury by use of protective cells or their products has been an area of intense research in the hopes of increasing long-term survival and kidney function (Donizetti-Oliveira et al., 2012; Furuichi et al., 2012).

Recently, the potential therapeutic use of mesenchymal stromal cells (MSC) has been investigated in many model systems. Based on the discovery of various properties of MSC to help in the repair of damaged tissues and to promote immunomodulatory functions, a great deal of promise has been invested in this cell type (Yagi et al., 2010; Hoogduijn et al., 2011; Shi et al., 2011; Singer and Caplan, 2011; Tögel, 2011). In animal experiment models of graft versus host disease (GvHD), skin transplantation, and in particular heart transplantation, MSC have been described as promoting protective effects (Bartholomew et al., 2002; Le Blanc et al., 2004; Maitra et al., 2004; Zhou et al., 2006; Eggenhofer et al., 2011). In a rat heart transplantation model, bone marrow derived donor- and recipient-type MSC administered concurrent to the time point of grafting were not able to prolong heart allograft survival or even led to accelerated rejection with concurrent low-dose Cyclosporin A treatment (Inoue et al., 2006). In contrast, pre-treatment with allogeneic MSC under mycophenolate mofetil (MMF) immunosuppression in the same rat transplantation model induced partial tolerance toward the transplanted organ, whereas syngeneic cells were less effective (Popp et al., 2009).

Beneficial effects of MSC on renal function were mostly described in models of acute kidney injury induced by temporary vessel ligation. In this experimental setup, MSC administration has been clearly shown to reduce kidney damage as measured by reduced serum creatinine and urea levels (Tögel et al., 2005; Semedo et al., 2007; Cao et al., 2010; De Martino et al., 2010; Morigi et al., 2010). In addition, we could very recently show in a rat renal transplantation model that repeated recipient MSC application was able to ameliorate damage following prolonged cold ischemia at early time points by reducing the expression of pro-inflammatory cytokines and infiltration by antigen-presenting cells (APC) in the grafted kidney (Hara et al., 2011). However, in this acute pre-clinical model the allograft survival was not improved. As these results fell short of our expectations, we have focused on reports in the heart model that indicated that allogeneic MSC under MMF immunosuppression might be more effective (Popp et al., 2009).

Here, we describe that the protocol which was successful in a heart transplant model cannot simply be transferred to kidney transplantation. Allogeneic MSC do not induce tolerance to the graft, but they actually worsen the outcome. We have found that the deleterious effects of both donor- and recipient-type MSC are related to the induction of humoral immune responses, associated infiltration of B cells, and increased C4d deposits attributed to complement activation in the allograft. We also found indications that the allogeneic MSC could lead to a pre-sensitization of the recipient to donor antigens as shown by the enhancement of donor-specific antibodies that could accelerate the pace of organ rejection instead of hindering it.

## MATERIALS AND METHODS

### ANIMALS

Adult male Dark Agouti (DA; MHC haplotype RT1^ av^; Harlan-Winkelmann, Borchen, Germany) and Lewis (LEW; MHC haplotype RT1^l^) inbred rats (Charles River, Sulzfeld, Germany) weighing approximately 250–300 g were maintained in the animal facility of the Charité Virchow clinic. All animal procedures were performed in accordance with the approval of the local authority for animal research procedures, the Landesamt für Gesundheit und Soziales, Berlin, Germany, and conformed to all relevant regulatory standards for animal research. The rats were anesthetized with inhaled isoflurane.

### MSC ISOLATION AND CULTURE

**FIGURE A1:**
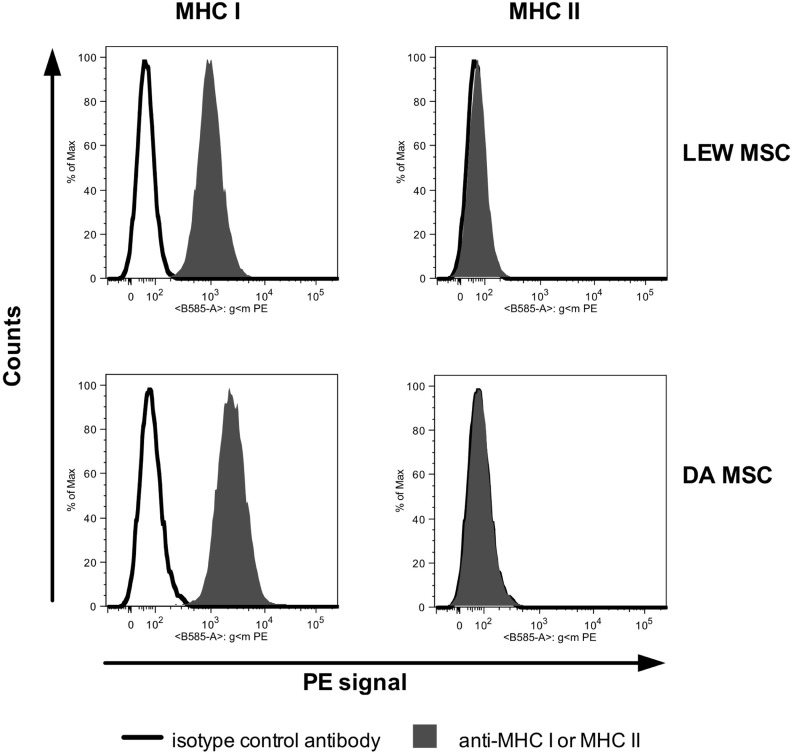
** FACS histograms of a representative labeling of LEW MSC (recipient-type) and DA MSC (donor-type) with antibodies against rat MHC I and MHC II are shown (gray filled curve) in comparison to labeling with the isotype control antibody (black bold line).** Both MSC types constitutively express MHC I, but not MHC II. Data were collected using a BD FACS Canto II flow cytometer analyzed using FlowJo software.

Mesenchymal stromal cells were harvested from bone marrow of femurs and tibias from adult male LEW or DA rats by centrifugation of the bone shaft as described elsewhere (Hara et al., 2011). MSC at passages 3–5 and a content of <5% CD45^+^ cells as confirmed by flow cytometry were used for all experiments described. MSC displayed a typical phenotype pattern: CD90^+^, CD73^+^, major histocompatibility complex (MHC) I^+^, intercellular adhesion molecule (ICAM)^+^, VCAM^-^, MHCII^-^, CD86^-^, and weak CD80^+^ as described elsewhere (Hara et al., 2011; see also Appendix **Figure A1**).

### KIDNEY TRANSPLANTATION

Donor kidneys were removed from DA rats and then perfused with and stored in University of Wisconsin (UW) perfusion solution (Charité, Berlin, Germany) at 4°C while the recipient animal was prepared. The total cold ischemic time was 35 ± 5 min. Following cross-clamping of the abdominal aorta and the inferior vena cava, the left kidney of the LEW recipient rat was removed. The DA kidney was transplanted orthotopically with an end-to-side aortic patch and performing an end-to-end venous anastomosis using 10-0 Prolene® (Ethicon; Johnson & Johnson Medical GmbH, Norderstedt, Germany) running sutures. The ureter was reconstructed by using an end-to-end anastomosis, performed by four discontinuous stitches with 10-0 Ethilon® (Ethicon). The total warm ischemic time of the graft during the attachment of the new kidney was approximately 15 min.

### EXPERIMENTAL DESIGN

**FIGURE 1:**
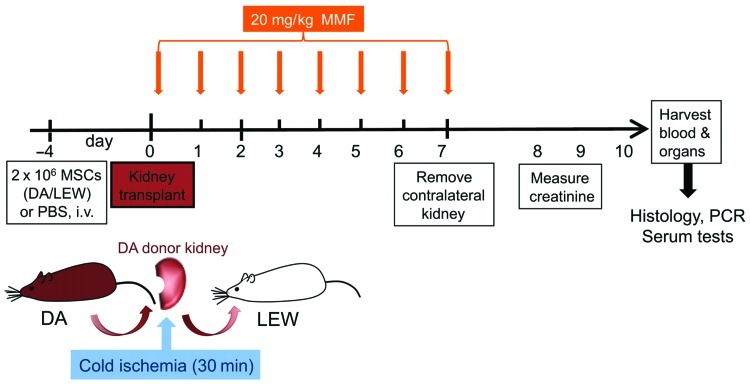
** Experimental flow chart of* in vivo* study design.** DA (DA, RT1^ av^) kidneys were harvested, perfused with UW solution and kept at 4°C before being transplanted orthotopically into Lewis (LEW, RT1A^l^) recipients. MMF treatment was given daily from day 0 to 7 at a dosage of 20 mg/kg body weight. Two million donor-type DA-MSC or recipient-type LEW-MSC or PBS controls were intravenously injected 4 days before transplantation with five to six animals in each group. Eight to ten days after transplantation, rats were humanely euthanized and tissues were harvested for further analysis by real-time PCR and immunohistology.

As outlined in **Figure 1**, 4 days prior to kidney transplantation, two million bone marrow-derived MSCs from DA or LEW rats, or phosphate buffered saline (PBS) as control was injected intravenously. The immunosuppressant mycophenolate mofetil (MMF/Cell Cept; Roche, Basel, Switzerland) was injected i.p. at a dosage of 20 mg/kg body weight as described in a study of MSC in heart transplantation (Popp et al., 2009) under mild isoflurane anesthesia daily for 7 days from the day of transplant (day 0). Transplanted rats were monitored daily for signs of illness due to rejection or side effects of the MMF treatment. The contralateral (right side) kidney was removed at day 7 after transplant. Signs of rejection appeared beginning at day 8 for all groups. Data were collected from five to six individual animals in each treatment group that were transplanted and treated independently with two to three transplantations performed per week (*n* = 5–6). Please note that the creatinine measurement is only shown for *n* = 4–6 animals per group as this data was not measured from one animal.

The rats were anesthetized with isoflurane and blood was collected from the aorta using a catheter (Venflon^ TM^ Pro 22GA; BD Biosciences, Heidelberg, Germany) into a serum collection tube (Vacutainer® SST II, 8.5 ml; BD Biosciences) with an additional blood drop placed onto a CREA Reflotron strip to measure creatinine levels using a Reflotron® Plus Clinical Chemistry analyser (Roche Diagnostics, Mannheim, Germany). After perfusing the transplanted kidney with cold saline, the grafted kidney and recipient spleen were collected for further analysis by PCR or immunohistochemistry.

### QUANTITATIVE REAL-TIME RT-PCR

Harvested organs were carefully cut into smaller pieces, immediately snap frozen in liquid nitrogen and stored at -80°C. Kidneys and spleens were thawed and homogenized before total RNA was extracted using the Nucleospin II RNA kit (Macherey-Nagel GmbH & Co.KG, Düren, Germany) and quantified using the Nanodrop 1000 device and v3.7.1 software (Peqlab, Erlangen, Germany). A reverse transcription reaction was performed using 3 µg total RNA in a total volume of 30 µl using the high capacity cDNA reverse transcription kit (Applied Biosystems/Life Technologies GmbH, Darmstadt, Germany) in an Eppendorf Mastercycler personal thermal cycler (Eppendorf, Hamburg, Germany) using the conditions 10 min at 25°C, 2 h at 37°C, and 5 s at 85°C as recommended by the manufacturer. Quantitative real-time PCR was performed using the Eppendorf realplex^2^ Mastercycler machine with a total reaction volume of 20 µl in 0.2 ml MicroAmp® Optical Tubes and strip lids (Applied Biosystems) for a total of 40 cycles. The PCRs for tumor necrosis factor α (TNFα), interferon γ (IFNγ), interleukin-6 (IL-6), CD25, and MHC class II were performed using TaqMan chemistry (TaqMan® Universal PCR Mastermix; Applied Biosystems), and for chemokine ligand (CCL) 21, IL-1β, and β-actin using SYBR® Green qPCR MasterMix Plus dTTP for SYBR® Assay ROX (Eurogentec, Seraing, Belgium). Primers and probes were synthesized by Metabion (Martinsried, Germany) with sequences given in **Table 1**. For ICAM-1, an assay on demand was used (Applied Biosystems). The specificity of the desired gene products was determined by melting-curve analysis. Expression of the housekeeping gene β-actin was used to normalize expression of the target gene within the test sample and the mean fold increase of the target gene in the test samples compared to the values in the kidneys or spleens of three naïve rats was calculated using the formula 2^-ΔΔCT^ (Livak and Schmittgen, 2001).

**Table 1:** Primer and probe sequences used for real-time RT-PCR analysis.

Gene	Forward primer	Reverse primer	Probe
TNFα	5′-tcg agt gac aag ccc gta gc-3′	5′-ctc agc cac tcc agc tgc tc-3′	5′-cgt cgt agc aaa cca cca agc aga-3′
IFNγ	5′-aac agt aaa gca aaa aag gat gca tt-3′	5′-ttc att gac agc ttt gtg ctg g-3′	5′-cgc caa gtt cga ggt gaa caa ccc-3′
IL-1β	5′-acc aaa aat gcc tcg tgc tgt ct-3′	5′-tgt tgg ctt atg ttc tgt cca ttg-3′	5′-acc cat gtg agc tga aag ctc tcc acc-3′
IL-6	5′-aac tcc atc tgc cct tca gga-3′	5′-ggc agt ggc tgt caa caa cat-3′	5′-ttt ctc tcc gca aga gac ttc cag cca-3′
CCL21	5′-cca tcc cag caa tcc tgt tc-3′	5′-cct cag ggt ttg cgc ata-3′	-
MHC class II	5′-ggt tga gaa cag caa gcc agt c-3′	5′-ggt gag gta agc cat ctt gtg g-3′	5′-tga gac cag ctt cct ttc caa ccc tga-3′
CD25	5′-cac agt ctg tgt acc aggaga acc t-3′	5′-cca cga agt ggt aga ttc tct tgg-3′	5′-cag gtc act gca ggg agc ccc c-3′
β-actin	5′-gta caa cct cct tgc agc tcc t-3′	5′-ttg tcg acg acg agc gc-3′	5′-cgc cac cag ttc gcc atg gat-3′

### IMMUNOHISTOCHEMISTRY FOR INTRAGRAFT CELLULAR INFILTRATION

Harvested organs were prepared for immunohistochemistry by first fixing the tissues with 2% paraformaldehyde (PFA; Sigma-Aldrich, Taufkirchen, Germany) for 2 h and were then transferred to 30% filter-sterilized sucrose (Calbiochem/Merck, Darmstadt, Germany) for 1 or 2 days before being embedded in Jung Tissue Freezing Medium (Leica, Nussloch, Germany) and stored at -80°C. Sections of kidney or spleen tissues 5–8 µm thick were prepared using a Leica CM3050S cryostat and mounted onto Superfrost Plus slides (R. Langenbrinck, Emmendingen, Germany). Slides were blocked with Dual Enzyme blocking reagent (Dako Deutschland GmbH, Hamburg, Germany) for 10 min and washed, followed by 1 h with Tris buffered saline (TBS)/Tween/1% Bovine serum albumin (BSA)/10% horse serum before the addition of specific monoclonal mouse anti-rat antibodies to a B cell marker (clone KiB1R; BMA Biomedicals, Augst, Switzerland), MHC class II (MHCII/RT1B; clone OX-6; BD Pharmingen, San Diego, USA), CD45 (clone OX-1; AbDSerotec, Düsseldorf, Germany), CD68 (clone ED1; AbDSerotec), T cell receptor (TCR; clone R73; Biolegend, San Diego, USA) or with IgG isotype-identical control antibody (clone MOPC; Biolegend) overnight at 4°C. The primary antibody was thoroughly washed before incubation with the Secondary Antibody (anti-mouse IgG (H + L)-biotin, rat absorbed (Vector, Burlingame, CA, USA) for 1 h at room temperature, followed by incubation with streptavidin/horseradish peroxidase (Streptavidin/HRP; Dako Deutschland GmbH) and then visualized using substrate (3-amino-9-ethylcarbazole; AEC)-solution (Dako Deutschland GmbH). Samples were counterstained with Harris’s hematoxylin to detect cell nuclei and embedded in Aquatex (Merck, Darmstadt, Germany). Images were obtained by light microscopy using a Zeiss Axioskop 40 microscope (Carl Zeiss MicroImaging GmbH, Göttingen, Germany) with three images captured from each slide and then analyzed in a blinded approach by three different independent investigators. Signal intensities were graded as scores between 0 and 3 (0 = no staining, 1 = weak staining, 2 = moderate staining, 3 = strong staining). The scores obtained were graphed and analyzed in GraphPad Prism v5. Additional slides were stained for 4 min with Harris’s hematoxylin, washed twice with water, counterstained for 2 min with Eosin (both from Sigma-Aldrich, Taufkirchen, Germany), washed again and embedded with Entellan® (Merck) to evaluate tissue integrity.

### C4d STAINING

Immunofluorescence techniques were used to evaluate complement staining using a polyclonal antibody to rat C4d (Hycult Biotech, Uden, The Netherlands) which was incubated overnight at 4°C followed by an Alexa Fluor® 488 donkey anti-rabbit IgG (H + L) antibody (Jackson ImmunoResearch Europe Ltd., Suffolk, UK) for 90 min and covered with a DAPI mounting medium (Dianova, Hamburg, Germany). Images were obtained by fluorescence microscopy using a Zeiss Axis Observer Z1 microscope. The total area of positive C4d staining in square pixels was quantified using the Columbus^™^ Image Data Storage and Analysis System v2.3.0 (Perkin Elmer, Waltham, USA).

### DETECTION OF DONOR-SPECIFIC ANTIBODIES

Thymocytes were isolated from naïve male DA rats (200–250 g body weight) and made into a single cell suspension in Dulbecco’s PBS (PAA, Pasching, Austria) by homogenization through a 40µm cell strainer (Falcon, Oxnard, USA), and frozen in 90% fetal calf serum (FCS; Biochrom AG, Berlin, Germany), 10% dimethylsulfoxide (DMSO; Sigma, Taufkirchen, Germany) until use. The cells were thawed, washed twice in RPMI (PAA) with 2 mM L-glutamine (Life Technologies, Darmstadt, Germany) 100 units/ml penicillin and 100 µg/ml streptomycin (both from Life Technologies), containing 10% FCS (Biochrom AG) and incubated in a 37°C humidified incubator with 5% CO_2_ for 2 h. Thymocytes were washed again in cold PBS containing 1% FCS, strained through a 40µm cell sieve to remove clumps, and counted using a Fuchs Rosenthal cell chamber before 0.5 million cells were distributed into each 1.4 ml flow cytometry tube (Micronic, Lelystad, The Netherlands) and incubated with the serum collected from the test rats (or a naïve LEW rat as control) diluted 1:10 with PBS and incubated for 45 min at 4°C with occasional vortexing. Cells were washed thoroughly before incubation with Goat-anti-Rat-Fab2-FITC secondary antibodies for anti-IgG or anti-IgM (STAR 69 and STAR 116F; both from AbDSerotec, Düsseldorf, Germany) for 30 min at 4°C, washed again and fixed with 1% PFA (Sigma) until FACS analysis. Flow cytometry was performed using the BD FACS Canto II (BD Biosciences, Heidelberg, Germany) and further analysis with FlowJo 8.8.5 Software (TreeStar Inc., Ashland, USA) was used to determine the geometric mean fluorescence intensity (MFI) of FITC labeling. Background staining was calculated for a naïve Lew serum sample and subtracted from the test rat values.

### STATISTICAL ANALYSIS

All data are presented as means ± SEM. Data were analyzed for statistical significance by one-way analysis of variance (ANOVA) with the Bonferroni post-test for differences between groups using GraphPad Prism v5 software. *P* values of <0.05 were considered significant.

## RESULTS

### NEGATIVE IMPACT OF MSC ON KIDNEY GRAFT FUNCTION

**FIGURE 2:**
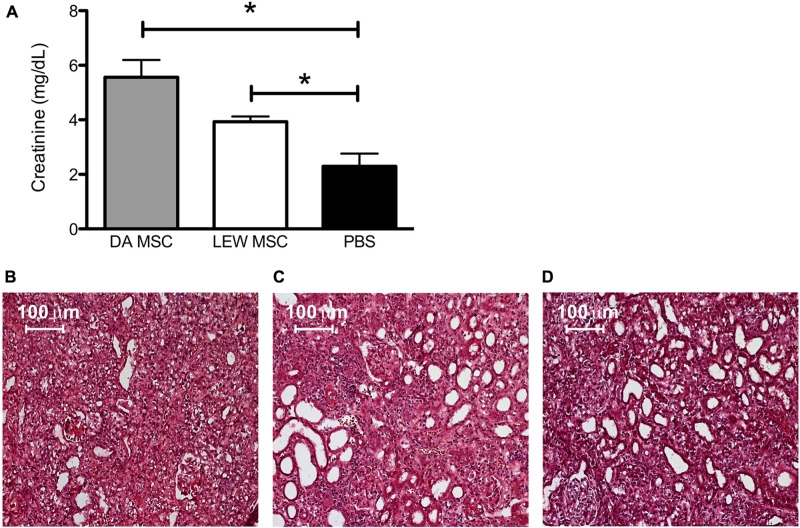
** MSC pre-treatment induces enhanced creatinine levels and impairs kidney cortex architecture.** Blood creatinine levels were measured 8–10 days after kidney transplantation and data are presented as mean ± SEM with 4–6 rats in each group** (A)**; *P < 0.05. Transplanted kidneys were then harvested, fixed with 2% PFA for 2 h, incubated in sterile 30% sucrose and embedded. Five to eight micron thick sections were stained with Harris’s hematoxylin followed by Eosin to evaluate tissue integrity. Representative images are shown for rats injected 4 days prior to transplant with **(B)** DA-MSC, **(C)** LEW-MSC, or **(D)** PBS. Scale bars represent 100 µm.

When serum creatinine levels were measured after removing the contralateral kidney, we found highest values in the DA-MSC-treated group which were significantly different to the PBS control group. Moreover, the creatinine values of the LEW-MSC-treated group were also significantly elevated, indicating reduced kidney function after injection of either type of MSC (**Figure 2A**). Hematoxylin-Eosin (HE) staining of cryosections corroborated these kidney function findings by indicating extreme destruction of the architecture of glomeruli and tubuli within the DA-MSC and LEW-MSC groups (**Figures 2B,C**) in comparison to the PBS-treated animals (**Figure 2D**).

### ENHANCED INFLAMMATION BY MSC IN RENAL ALLOGRAFTS

**FIGURE 3:**
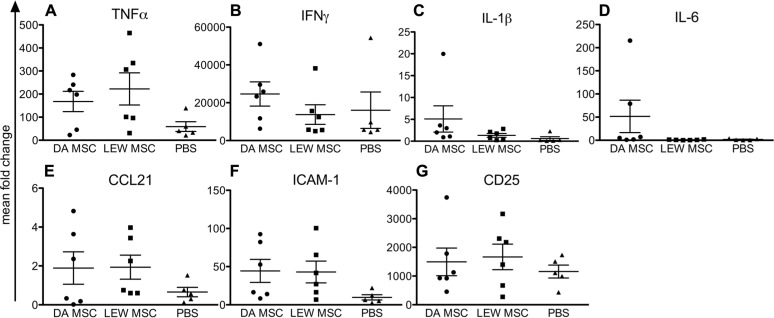
** Inflammation within the kidney grafts following MSC pre-treatment measured by intragraft gene expression analysis.** Quantitative real-time PCR was performed on kidney grafts harvested 8–10 days after transplant. After expression of the target gene was normalized to the housekeeping gene β-actin, the mean fold increase of the target gene in the test samples compared to the values in the kidneys of three naïve rats was calculated using the formula 2^-^^Δ^^Δ^^CT^ for **(A)** TNFα, **(B) **IFNγ, **(C) **IL-1β, **(D) **IL-6, **(E) **CCL21, **(F) **ICAM-1, and **(G) **CD25. Data are presented as the mean ± SEM of the mean fold change from five to six transplanted rats per group from PCR analyses performed in duplicate.

Kidney grafts were analyzed by quantitative PCR for their expression levels of inflammatory cytokines, chemokines, and cellular markers compared to naïve rats as shown in **Figure 3**. Although we could not find any significant differences between the expression level for all tested markers due to individual variations, it was obvious that PBS-injected animals in general showed lower values, especially for TNFα (**Figure 3A**), CCL21 (**Figure 3E**), and ICAM-1 (**Figure 3F**) when compared to both MSC-treated groups (DA- and LEW-MSC). IFNγ, IL-1β, IL-6, and CD25 mRNA expression was rather comparable between all groups (**Figures 3B–D,G)**.

**FIGURE 4:**
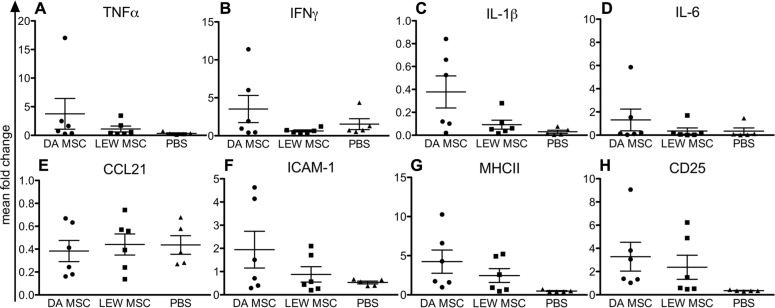
** Elevated immune cell activation in rat spleens following MSC pre-treatment measured by gene expression analysis.** Quantitative real-time PCR was performed on spleens harvested 8–10 days after kidney transplantation. After expression of the target gene was normalized to the housekeeping gene β-actin, the mean fold increase of the target gene in the test samples compared to the values in the spleens of three naïve rats was calculated using the formula 2^-^^Δ^^Δ^^CT^ for **(A)** TNFα, **(B) **IFNγ, **(C) **IL-1β, **(D) **IL-6, **(E) **CCL21, **(F) **ICAM-1, **(G) **MHCII, and** (H) **CD25. Data are presented as the mean ± SEM of the mean fold change from five to six transplanted rats per group from PCR analyses performed in duplicate.

Analyzing the mRNA expression levels in recipient spleens, we generally detected low expression levels for nearly all tested markers, except for CD25 (**Figure 4**). In addition, both MSC-treated groups displayed higher CD25 mRNA expression levels relative to the PBS control group (**Figure 4H**). Notably, values within the DA-MSC-injected group were more variable between the single recipients. Although not significant, more animals per group with higher expression levels were detected for the DA-MSC group and especially for the markers IL-1β (**Figure 4C**), ICAM-1 (**Figure 4F**), and MHCII (**Figure 4G**).

### IMPACT OF MSC ON CELLULAR INTRAGRAFT INFILTRATION

**FIGURE 5:**
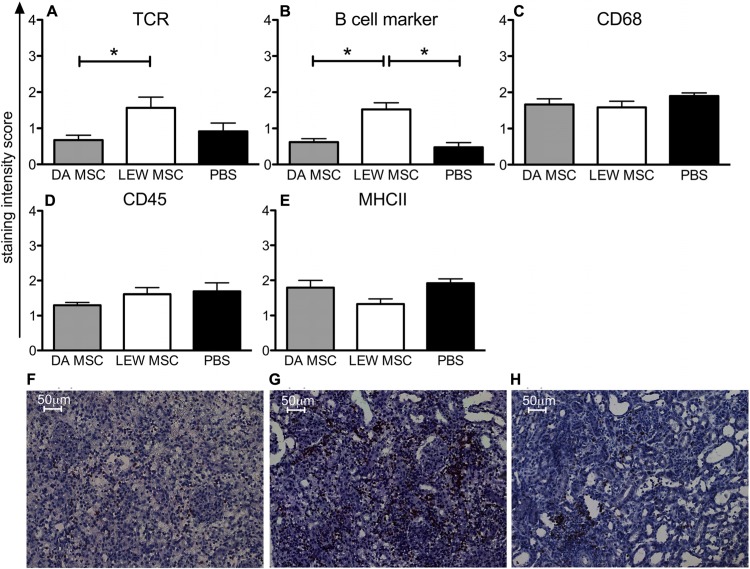
** Enhanced intragraft accumulation of, T and B cells following MSC pre-treatment measured by immunohistology.** Five to eight micron thick sections of transplanted kidneys were labeled with specific monoclonal mouse anti-rat antibodies to **(A)** TCR, **(B)** B cell marker, **(C)** CD68, **(D)** CD45, and **(E) **MHCII overnight at 4°C. A biotin-conjugated secondary antibody and streptavidin/HRP were applied before visualization with the substrate solution and counterstaining with Harris’s hematoxylin. Staining scores are given as mean ± SEM for triplicate slides with five to six rats in each group; *P <0.05. Representative images of B cell staining are shown for rats injected 4 days prior to transplant with **(F)** DA-MSC, **(G)** LEW-MSC, or **(H)** PBS. Scale bars represent 50µm.

Kidney grafts were analyzed by immunohistological staining for their cellular infiltration pattern at post-operative days 8–10 by staining with antibodies to the major subsets of immune cells. In **Figure 5**, the summarized data of staining scores for B cells, T cells, CD68^+^ macrophages, CD45^+^ leucocytes, and MHCII^+^ APC are shown. Significant differences between the experimental groups treated with LEW-MSC compared to DA-MSC-treated and PBS control animals were detected regarding the scores for TCR-positive cells (**Figure 5A**) and B cell marker-positive cells (**Figure 5B**). Surprisingly, higher values were detected for the LEW-MSC-treated group relative to the DA-MSC- and also the PBS-treated group as illustrated in representative images (**Figures 5F–H**). For all the other markers tested; CD68, CD45, and MHCII, the scores were comparable (**Figures 5C–E**).

### DEPOSITION OF COMPLEMENT FACTOR C4D IN THE KIDNEY CORTEX

**FIGURE 6:**
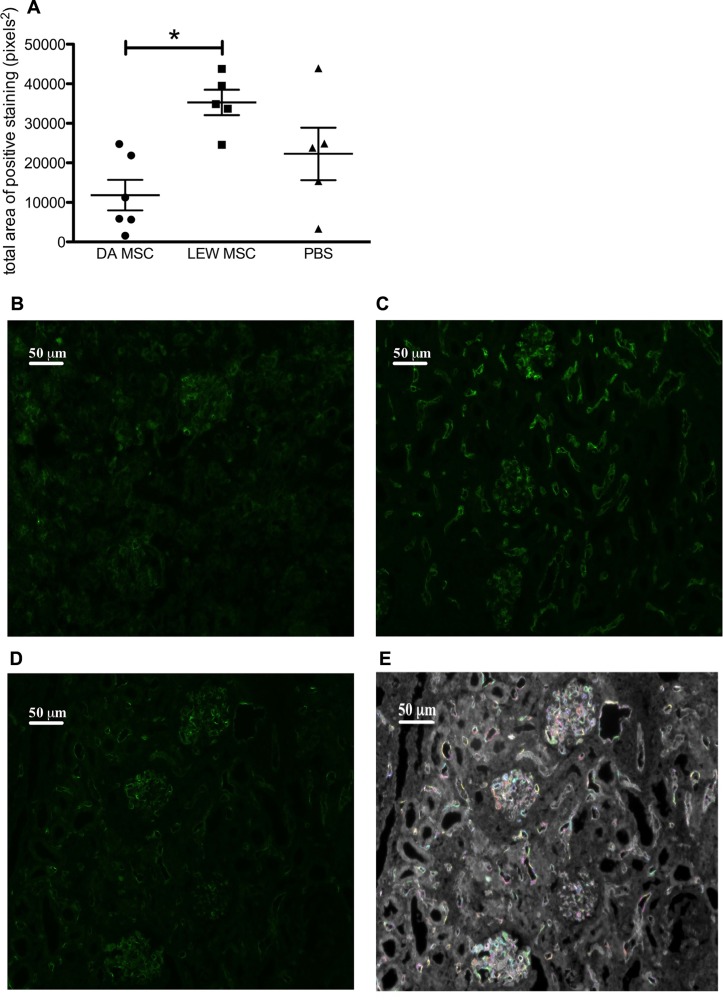
** Amplified C4d deposits in transplanted kidneys following MSC pre-treatment.** Immunofluorescence labeling was performed on sections prepared from transplanted kidneys using a polyclonal antibody to rat C4d incubated overnight followed by detection with an Alexa Fluor^®^ 488 donkey anti-rabbit IgG (H + L) secondary antibody. **(A) **Images obtained by fluorescence microscopy were evaluated using the Columbus^™^ Image Data Storage and Analysis System to quantify C4d (green fluorescence) labeling. Data are given as mean ± SEM for total area of positive “spots” in pixels^2^ from duplicate images from five to six rats per group; *P < 0.05. Representative images of C4d labeling are shown for rats injected 4 days prior to transplant with **(B)** DA-MSC, **(C)** LEW-MSC, or **(D)** PBS. An example of the spot identification using Columbus^™^ software is shown **(E) **with various colors indicting quantified spots for the same PBS-injected animal image. Scale bars represent 50µm.

**FIGURE A2:**
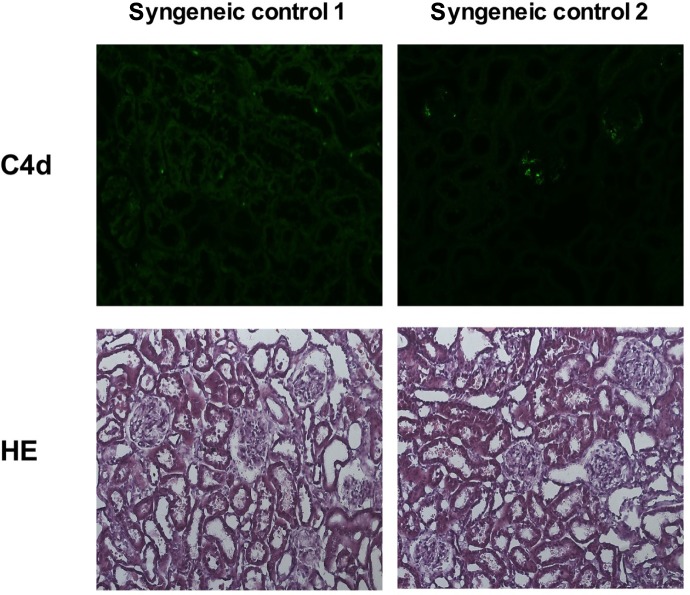
**Representative images of kidney cortex tissue of two animals 3 days after syngeneic kidney transplantation are shown.** Cryostat sections of the kidney tissue were stainedwith an anti-C4d antibody (upper row) or with HE (lower row); original magnification × 20.Both rats show a normal kidney cortex architecture and lack specific C4d signal.

To evaluate whether humoral mediated responses might contribute to the poorer graft function of MSC-treated rats, we performed immunofluorescence staining for C4d deposits. The staining intensities of the fluorescence signal on microscopic images of all samples were quantified by a specific algorithm of the Columbus^™^ Image Data Storage and Analysis System and the total area of positive staining (pixels^2^) was calculated for all treatment groups (**Figure 6A**). Representative images of the C4d staining for all treatment groups are shown (**Figures 6B–D**) as well for the quantification method (**Figure 6E**). A control staining performed using a transplanted syngeneic kidney demonstrated the absence of C4d deposits when rejection was not induced (please refer to Appendix **Figure A2**). It is apparent that LEW-MSC-treated animals express higher levels of C4d in the kidney cortex area then DA-MSC-treated rats but without significant differences between both treatment groups and the PBS-injected group.

### INDUCTION OF DONOR-SPECIFIC ANTIBODIES BY MSC APPLICATION

**FIGURE 7:**
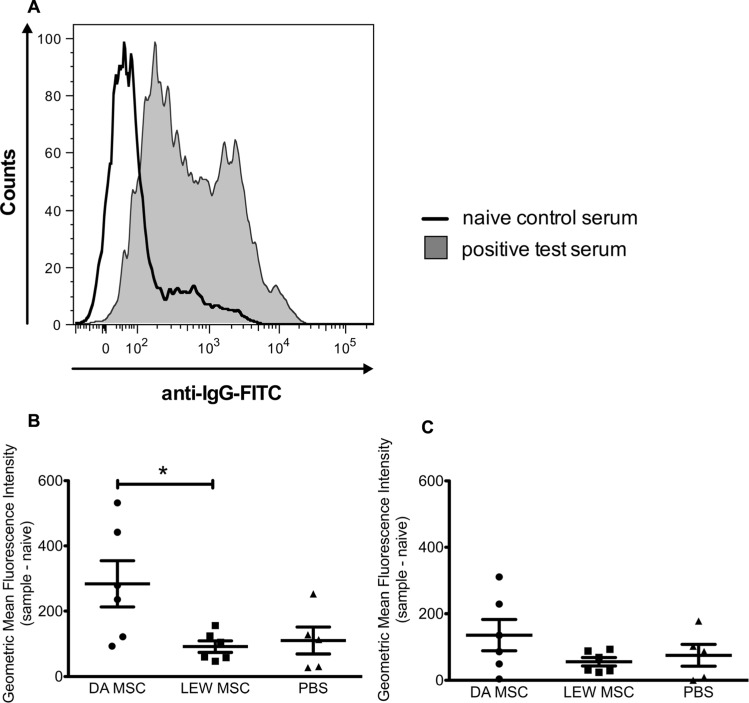
** Enhanced levels of donor-specific antibodies in serum following donor-specific MSC pre-treatment.** Serum collected from transplanted rats at the time of harvest was evaluated for the presence of IgG and IgM donor-specific antibodies. **(A) **A representative staining histogram is shown for a naïve control serum (black solid line) and a positive test serum (filled gray curve). Data were collected using a BD FACS Canto II flow cytometer and the geometric MFI was determined. Background values for a naïve animal were subtracted from test rat serum values. Data are presented for **(B) **IgG and **(C) **IgM donor-specific antibodies as the mean ± SEM of the geometric MFI from five to six transplanted rats per group; *P < 0.05.

Sera of all LEW recipient rats were screened for the presence of donor (DA)-specific antibodies at the time point of graft harvest using a flow cytometry based assay with isolated thymocytes. The geometric MFIs were calculated by subtracting the value of a naïve rat from the value of all kidney transplant recipient rats and a representative histogram of the fluorescence staining is shown in **Figure 7A**. As shown in the summarized data a distinct and significantly higher MFI for donor-specific IgG antibodies was measured for the DA-MSC-treated group in comparison to the LEW-MSC and PBS group (**Figure 7B**). IgM antibody values were only marginally enhanced in the DA-MSC-treated group (**Figure 7C)**.

**FIGURE 8:**
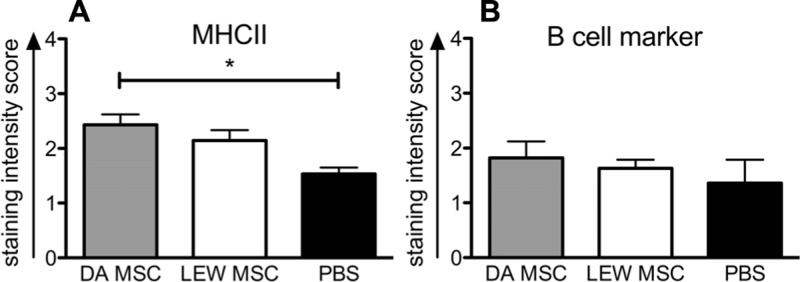
** Enhanced detection of MHCII-positive cells in rat spleens following MSC pre-treatment measured by immunohistology.** Spleens were harvested at 8–10 days after kidney transplant and cryosections were labeled with specific monoclonal mouse anti-rat antibodies to B cell marker, MHCII, or with the isotype control antibody overnight at 4°C. A biotin-conjugated secondary antibody and streptavidin/horseradish peroxidase were applied before visualization with the substrate solution and counterstaining with Harris’s hematoxylin. Triplicate images were captured from each slide and the intensity of positive brown staining for **(A)** MHC II or **(B)** B cell marker was analyzed in a blinded approach on a scale of 0–3. Scores are given as mean ± SEM for triplicate slides with five to six rats in each group; *P < 0.05.

Whether the higher levels of IgG donor-specific antibodies within the DA-MSC-treated group correlated with higher B cell activity in the spleen was analyzed by immunohistological staining of tissue sections with B cell- and MHCII-specific antibodies (**Figure 8**). The staining intensity score for MHCII was significantly enhanced in the DA-MSC transplant group compared to the PBS-treated control (**Figure 8A**). A trend toward an increase in MHCII was also observed for the LEW-MSC group. In contrast, B cell staining scores were nearly equal for all treatment groups (**Figure 8B**).

## DISCUSSION

In the present study, we investigated the effect of the donor-type MSC pre-treatment on the modulation of inflammation and rejection responses in an acute rat renal transplantation model of high MHC disparity with concomitant immunosuppression.

In our clinically relevant transplant model, we found that in contrast to our expectations, the application of either donor- or recipient-type MSC 4 days before kidney grafting resulted in the induction of increased signs of inflammation and higher levels of cellular infiltration, especially of B cells, at the time point of rejection. This was combined with C4d deposits within the glomeruli and the peritubular capillaries. In addition, when donor-type MSC were applied, significantly higher donor-specific IgG-antibody levels were induced, in contrast to the application of recipient-type MSC. These data lead us to the conclusion that donor-type MSC administration before kidney transplantation causes enhanced humoral rejection processes.

Our data in a renal transplant model are in contrast to the clear beneficial effects of the day-4 MSC application in a rat model of heterotopic heart transplantation with the same MMF immunosuppressive regimen (Popp et al., 2009). We neither see a prolonged graft survival, nor the development of partial tolerance. Discrepancies might be caused by differences in the experimental parameters including the selected rat strain combination, and the fact that while the heterotopic heart is not required for survival, our model requires the transplanted kidney to function.

Renal grafts were rejected between days 8 and 10 regardless of whether the groups were pre-treated with MSC or a PBS control. The overall condition of the animals was extremely poor in the donor-type MSC-treated group, and higher creatinine levels were measured at the time point of rejection. This was also reflected by the observation of histological signs of destruction which damaged the typical renal cortex architecture of glomeruli and tubuli and visible interstitial cellular infiltration in HE staining.

Analyzing the degree of cytokine and cellular marker expression within the grafted kidneys, we found not significantly changed gene expression levels between the MSC-treated and the PBS-treated control group. However, most animals in both MSC-treated groups tended toward higher mRNA expression levels for the pro-inflammatory cytokine TNFα and the chemokine CCL21 as well as cellular markers of immune cell activation (e.g. ICAM-1, CD25). Therefore, the protective effect of MSC by reducing signs of inflammation at early time points after kidney transplantation we recently described (Hara et al., 2011) seems to be undetectable in a later phase of the rejection process. In this former *in vivo* study, we saw significant effects using a higher number of cells which were injected at multiple time points both before and after transplantation. In addition, the type of immunosuppressive treatment (Cyclosporine A instead of MMF) might influence the difference in MSC effectiveness observed.

Splenic mRNA levels for IL-1β and for ICAM-1 and MHCII also trended toward an increase in the donor-type MSC-treated group, indicating signs of sensitization against the donor type cells and induced immunogenicity. Whether the observed marginally higher CD25 expression in both MSC-treated groups is related to expansion of regulatory T cells or activation of conventional T cells remains unclear.

Our analysis of graft infiltrating cell subsets gave rise to interesting and surprising findings. We observed that B cells were more abundant within the grafts pre-treated with recipient-type MSC compared to the PBS control group. T cell and macrophage infiltration were not significantly different between the PBS and both MSC groups. Why the donor MSC-treated animals do not display the same high B cell staining is unclear. One explanation might be that the process of rejection and organ damage is even more accelerated over the same time frame in this group and cells had already disappeared from the allograft at days 8–10. Hints for stronger organ damage in the donor MSC-treated group were clearly seen in the HE histology and additionally resulted in higher blood creatinine levels. Whether the higher B cell infiltration within the kidneys and the higher degree of graft destruction is caused by enhanced IL-6, as described in rat kidney transplantation models with low weight grafts (Gong et al., 2009) was examined. However, we detected neither significantly elevated mRNA expression levels for IL-6 in the grafted kidneys, nor higher levels of circulating IL-6 in the serum of MSC-treated animals at the time point of rejection (data not shown). Future studies could clarify if MSC cause a rise in systemic IL-6 levels soon after they are injected which decreases over time.

It is known that B cells are an important immune cell subset with antigen-presenting capacity in renal graft rejection. B cell involvement is in general characterized by intragraft B cell infiltration, C4d deposition and circulating donor-specific antibodies (Barnett et al., 2011). In humans, about 5–7% of the kidney transplant patients develop acute humoral rejection (Takemoto et al., 2004). Therefore, we had a closer look into the detection of complement factor deposits and the circulation of donor-specific antibodies. Animals that were treated with recipient-type MSC have significantly higher levels of C4d deposits and more infiltrated B and T cells, when compared to the donor-type MSC group.

The analysis of the donor-specific antibody levels demonstrated a detectable IgG response in all animals which we attribute to the transplant of a strongly mismatched kidney. However, IgG levels were significantly higher in the donor MSC-treated group. These results clearly demonstrate the sensitization in recipients to the donor-type antigen resulting in a more accelerated rejection process. Our observations are in agreement with evidence from other groups demonstrating recognition of MSC by the adaptive immune system (Crop et al., 2011) or even sensitization of the recipient (Nauta et al., 2006; Sbano et al., 2008). Another group has recently published that i.v. injection of allogeneic MSC provoked the generation of allo-antibodies and that repeated injections reduce the survival of injected allogeneic MSC (Schu et al., 2011). However, it still remains unclear how this may interfere with their potential immunomodulatory effects in pre-clinical or clinical trials (Griffin et al., 2010; Hoogduijn et al., 2011).

The first clinical study in kidney transplantation with autologous MSC treatment was reported by Perico et al. (2011) as a safety and feasibility study, but with limited success. Other groups are preparing to set up clinical trials using autologous or even allogeneic MSC as described in a recent review (Roemeling-van Rhijn et al., 2012). Although in solid organ transplantation new treatment strategies are essential, our results from the pre-clinical rat kidney transplantation model advise that MSC administration may not be optimal in all types of solid organ transplants and also that the specific treatment regimen might be crucial for graft success. Contrary data have also been published for the rat heart transplantation model, with either accelerated rejection (Inoue et al., 2006) or prolonged graft survival (Popp et al., 2008) obtained depending on the experimental approach. Therefore, the time point of injection, number of cells applied and the type of immunosuppressive treatments used seem to be important parameters influencing the success of the MSC treatment. A recent study using autologous MSC as a replacement for induction therapy in living, related kidney transplants (Tan et al., 2012) demonstrated reduced acute rejection, faster recovery of renal function and reduced opportunistic infections. Whereas, another group observed prolonged graft survival by a Treg-dependent mechanism in a mouse model of kidney transplantation where they applied a pre-treatment of animals with syngeneic MSC (Casiraghi et al., 2012), indicating that the proper time point for MSC administration is still up for debate. Nevertheless, based on conflicting results in pre-clinical studies caution must be exerted in order to avoid adverse effects in future clinical studies.

Though the administration of whole MSC may lead to adverse effects, it is possible that many of the positive effects published in earlier studies could be due to paracrine modes of MSC action. As many researchers do not believe that MSC act to improve regeneration by differentiating into cells of the target organ to exert their effects (Tögel, 2007; Wise and Ricardo, 2012), rather that they might work by secreting paracrine factors or microvesicles (Bruno et al., 2009; Gatti et al., 2011; Ratajczak, 2011; Tetta et al., 2011), we would suggest that further studies focus on investigating the positive protective effects of MSC in organ regeneration without the risks of injecting whole cells. This approach would also alleviate concerns related to the possible malignant outgrowth of MSC injected into a patient subjected to long-term immunosuppression.

## Conflict of Interest Statement

The authors declare that the research was conducted in the absence of any commercial or financial relationships that could be construed as a potential conflict of interest.
